# Relation Orientation and Ageism: A Cross-Cultural Comparison Between Chinese and Americans

**DOI:** 10.1093/geroni/igaa057.3109

**Published:** 2020-12-16

**Authors:** Xin Zhang

**Affiliations:** Peking University, Beijing, China

## Abstract

Attitudes toward older adults were negatively associated with ageism. However, whether this association is universal or cultural specific remained unknown. On the basis of well-documented cultural difference in relation orientation between westerners and easterners, this study aimed to investigate whether participants of different cultural background would show different association between ageism and attitudes toward close vs. non-close older adults in a sample of 211 Chinese (Mean age = 33.27) and 241 American (Mean age = 34.56) younger adults. Multiple regressions were conducted, and as expected, attitudes toward older adults (of different relation orientation) were found to be associated with ageism differently in two cultures. For American participants, attitudes toward both close and non-close older adults significantly correlated with ageism, while only attitudes toward close older adults were significant predictors of ageism in Chinese sample. This result had important implications for understanding and intervening ageism with people of different culture background.

